# Living in a risky world: the onset and ontogeny of an integrated antipredator phenotype in a coral reef fish

**DOI:** 10.1038/srep15537

**Published:** 2015-10-30

**Authors:** Maud C.O. Ferrari, Mark I. McCormick, Bridie J. M. Allan, Rebecca Choi, Ryan A. Ramasamy, Jacob L. Johansen, Matthew D. Mitchell, Douglas P. Chivers

**Affiliations:** 1 Department of Biomedical Sciences, WCVM, University of Saskatchewan, SK, Canada; 2 ARC Centre of Excellence for Coral Reef Studies, and College of Marine & Environmental Sciences, James Cook University, Townsville, QLD, Australia; 3 Department of Biology, Macalester College, St. Paul, Minnesota, USA; 4 Whitney Laboratory for Marine Bioscience, University of Florida, St. Augustine, Florida, USA; 5 Department of Biology, University of Saskatchewan, SK, Canada

## Abstract

Prey individuals with complex life-histories often cannot predict the type of risk environment to which they will be exposed at each of their life stages. Because the level of investment in defences should match local risk conditions, we predict that these individuals should have the ability to modulate the expression of an integrated defensive phenotype, but this switch in expression should occur at key life-history transitions. We manipulated background level of risk in juvenile damselfish for four days following settlement (a key life-history transition) or 10 days post-settlement, and measured a suite of physiological and behavioural variables over 2 weeks. We found that settlement-stage fish exposed to high-risk conditions displayed behavioural and physiological alterations consistent with high-risk phenotypes, which gave them a survival advantage when exposed to predators. These changes were maintained for at least 2 weeks. The same exposure in post-settlement fish failed to elicit a change in some traits, while the expression of other traits disappeared within a week. Our results are consistent with those expected from phenotypic resonance. Expression of antipredator traits may be masked if individuals are not exposed to certain conditions at key ontogenetic stages.

Predation is a pervasive selective force known to profoundly alter many aspects of a prey’s biology, including its behaviour, morphology and even life history; the literature is rife with various examples of prey altering their biology to decrease their risk of being captured by predators^1^,^2^,^3^,^4^. For some species, the population-wide phenotypic variation in antipredator adaptations have stemmed from selective forces that have left high-risk populations defended, and low-risk population less defended. For instance, oceanic stickleback (*Gasterosteus aculeatus*) that entered freshwater and found themselves in lakes without fish predators showed considerable loss of protective armour while those occupying lakes with fish predators maintained their protective armour^5^,^6^. In guppies *Poecilia reticulata*, individuals from high-risk streams have a duller colour than their low-risk counterpart, an adaptation that decreases their conspicuousness to predators^7^,^8^. Females from high-risk streams also preferentially choose duller males, while their low-risk counterparts prefer brightly coloured ones^9^. This ensures that their offspring also share the colour morphotype that is beneficial in their respective environments. These types of genetically-driven adaptations usually evolve as a response to high predation pressure that is consistently selective and sustained over evolutionary timescales^10^,^11^.

For many species, the predation risk experienced by individuals may not be strong enough, unidirectional enough, or sustained enough to lead to a cascade of fixed (genetic, non-plastic) antipredator adaptations^12^. Indeed, for the most part, predation pressure is highly variable in space and time^13^,^14^. A number of prey species have been shown to respond to predation risk only when a threat is detected. For instance, individuals may alter the location and timing of foraging to decrease their likelihood of encountering predators^3^. Upon detection of predation cues, some prey have the ability to develop protective morphological traits, such as protective spines or an increase body depth to escape gape-limited predators^15^,^16^. For others, the adaptation will stem from altering the timing of life-history switch points to decrease the predation pressure on a particular life stage^2^. For instance, amphibians can hatch and/or metamorphose earlier or later after detecting egg- or larval-oriented predators^17^,^18^. Due to their costs, these adaptations are only displayed when a risky situation is detected. This phenotypic plasticity is thus adaptive in that it allows prey to maximize the trade-off between costly antipredator adaptations and fitness-related activities, such as foraging or mating. However, individuals maintaining this plasticity also endure a cost: the cost of plasticity^19^,^20^.

Individual prey can either evolve in predetermined high- vs low-risk habitats and display fixed antipredator defences, or endure such a variable risk regime in their lifespan that plasticity in the expression of antipredator adaptation is the best option. For others, species-specific dispersal patterns may prevent individuals from either having a cross-generational consistent environment (similar to that of the parents) or from predicting the type of environment in which they will find themselves. However, the type of environment in which they ultimately settle could be constant for the rest of their lifespan^2^,^21^. This is likely the case for prey having a bi- or tri-partite life history, where dispersal is not under parental control. For instance, many aquatic species will release their eggs in the ocean, where currents will disperse them. However, after the pelagic larvae settle into a benthic lifestyle, the community of predators experienced by the individuals might be relatively constant. Hence, these individuals cannot benefit from having a fixed antipredator strategy, as individuals may find themselves at a selective disadvantage if the fixed defensive phenotype does not match the type of environment in which they live. On the other hand, maintaining the ability to display different defensive phenotypes may be beneficial, but these individuals may endure the costs of maintaining this unneeded plasticity. In such situations, one would predict that individuals may have life-history options with regards to their level of investment in defences, similar to the life-history options available to species with regards to context-specific alternative reproductive or foraging strategies^22^.

Beyond the changes initiated by prey after encountering predators, recent evidence suggests that living in a high-risk environment, irrespective of any predator-specific cues, can alter the phenotype of prey^23^. Brown *et al.*^24^ demonstrated that both fish and amphibian larvae exposed multiple times per day for four consecutive days to chemical cues from damaged conspecifics (high-risk environment), would start displaying a fright response to any novel chemical cues (i.e., a neophobic response). This phenotype is completely absent in conspecifics maintained under low-risk conditions. A similar treatment of juvenile coral reef fishes affects the way in which prey categorize predators and non-predators^25^. Moreover, short-term changes in background risk influences behavioural lateralization, with high-risk individuals being more strongly lateralized than low-risk individuals in a standard detour test^26^. This is a rather surprising result, as lateralization is not typically thought of as a plastic trait, and four days is an extremely short timeframe to observe such changes.

Despite the wealth of studies on the ability of prey from different taxa to respond to predation risk, there is still a gap in our comprehensive understanding of factors that drive the expression and plasticity of antipredator responses in most taxa, in particular taxa with complex life histories. Thus, this study was designed to answer two questions: (1) does exposure to high or low background levels of risk lead to the expression of differing, integrated antipredator phenotypes, and if so, do they provide measurable survival benefits with different predators? (2) If a risk-related phenotype exists, is its expression linked to the ontogeny of the animal? We used settlement-stage damselfish to answer these questions, as the settlement of the fish onto coral reefs represent a major life-history transition, but also a predation-induced bottleneck, as 60–90% of juveniles will be depredated while settling^27^. Based on the hypothesis that exposure to high-risk conditions will turn on a prey’s antipredator phenotype, we predicted that changes would be observed in a number of behavioural and physiological endpoints, specifically that high-risk damselfish would display vigilance towards unknown cues, show a greater degree of behavioural lateralization, have a lower latency to initiate a C-start, and maintain a higher basal metabolic rate than low-risk fish would. If those traits confer survival benefits, we predicted that high-risk fish should survive better than low-risk fish during predator encounters. We also hypothesized that the appearance of an integrated antipredator phenotype should be linked to settlement (a major life history switch point), where individuals must rapidly adopt a behaviour and phenotype that suits their new environment. Consequently, we predicted that either the amplitude and/or duration of the expression of those traits should be lower if the presentation of novel environmental conditions were temporally mismatched with the timing of their life-history transition.

## Methods

All work carried herein was done in accordance with James Cook University ethical guidelines and approved by the JCU ethics committee.

### Test species

The whitetail damselfish, *Pomacentrus chrysurus*, is a common coral reef fish in the Indo-Pacific region, typically associated with coral rubble in shallow reef waters. It has a bipartite life history typical of many reef fishes, with a planktonic larval stage lasting ~20–25 days, before young fish recruit to coral reefs and transition to benthic juveniles that are highly territorial. This transition represents a major and rapid change in the physiology, sensory biology and morphology for most fishes, as they change forms from one suited to an open ocean existence to one better suited to a colourful, structurally complex benthic habitat^28^. This transition involves a severe population bottleneck, with more than 60% of individuals succumbing to predation within 1–2 days of settlement to the reef 27. This mortality can be highly selective^29^,^30^, though the directionality of the selection can depend on the predator population in the immediate vicinity^31^. These results highlight the importance of predation in structuring these communities. Indeed, juveniles are vulnerable to a diverse range of predators that use a variety of feeding modes from ambush (lizardfish *Synodus dermatogenys*) to pursuit (moonwrasse *Thalassoma lunare*). These predators can be routinely observed to consume juveniles that venture too far from shelter.

Newly metamorphosed, settlement-stage juveniles of the whitetail damselfish were collected overnight using light traps moored in open water around Lizard Island (14’40° S, 145’28° E), in the northern Great Barrier Reef, Australia in November and December 2013. Adult predatory moonwrasse and lizardfish were captured from a lagoon using barrier nets, kept in flow-through tanks and fed daily with squid pieces.

### Experimental protocol

To answer our first question, we maintained newly-caught juveniles under conditions of high or low risk for 4 days and tested for differences in a series of variables (physiological, behavioural and ecological) that would make-up a general antipredator phenotype: a) their *expression of vigilance or antipredator behaviour* (measured as change in foraging and activity) when exposed to the odour of an unknown predator; b) their *degree of behavioural lateralization* in a detour test; c) their differences in escape performance, measured as the *latency of initiate a C-start (escape) response* when detecting a looming stimulus; d) their metabolic response, quantified as the *basal metabolic rate* (BMR) and the *latency to reach BMR;* e) their *survival*, measured as the proportion of fish alive after a 22-h trial in a mesocosm containing either a lizardfish (ambush predator) or a moonwrasse (pursuit predator).

**Figure 1:**
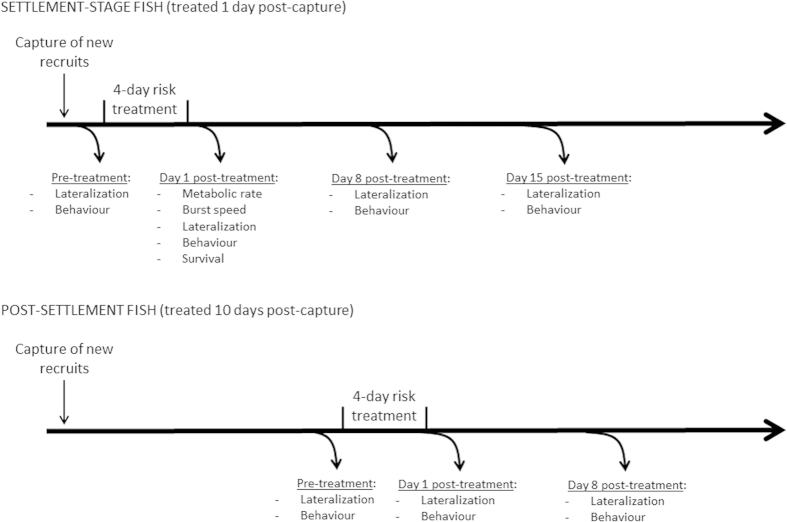
Summary of the nature and timing of the tests performed in our study. Fish were only used once and for a single variable.

To answer the second question, we indirectly compared the onset and maintenance of *lateralization* and *vigilance* between newly-caught fish (i.e., settlement-stage fish) and fish maintained in low-risk conditions in the laboratory for 10 days (post-settlement fish). We chose those two variables as they allowed us to collect enough data within the time-frame imposed by the experimental design. The comparison is indirect, because of the many factors that may confound a direct comparison between settlement-stage and post-settlement fish. Inherent differences in age, size, weight, etc. may affect response intensities through time, so we compared the *response pattern* (mostly, presence or absence of responses) between the two groups of fish. To eliminate the possible temporal confound from the sequential nature of our tests (if all the settlement-stage fish were tested first, and all the post-settlement fish were tested last), we continually collected new recruits over the testing period and ensured all tests temporally overlapped. The fish were arbitrarily allocated to be tested for a given variable and were only used once in the experiment. The timing of all procedures is summarized in Fig. 1.

### Creating high and low background levels of risk

To create a high-risk environment that would not provide specific information about the diversity, density and predator species causing it and subsequently bias the response towards a specific predator type, we decided to use non-predator specific general risk cues. Injured conspecific cues (hereafter alarm cues) are chemicals that innately elicit an overt antipredator response when detected by nearby conspecifics^32^. Given that these cues are located in the skin of prey and thus can only be released in the water column via mechanical damage to the skin (which would usually occur during a predator attack), they represent a reliable indicator of risk and mediate many antipredator adaptations in aquatic species^32^. Many damselfish species, including the whitetail damselfish, are known to possess and respond to cues from injured conspecifics^33^,^34^.

Following their capture, juvenile damselfish were immediately taken to the laboratory and placed in groups of 10 in a series of 3-L flow-through plastic aquaria with a flow rate of approximately 3 L/h. The fish were fed ad libitum with newly hatched brine shrimp 3 times per day. We left them to acclimate for 24 h before starting the experimental treatment. Fish were then exposed to high- or low-risk conditions by introducing a solution of alarm cues (high risk) or a seawater control (low risk) into the tanks 3 times per day for 4 days. Half the fish received the high-risk treatment while the remainder of the fish received the low-risk treatment. The alarm cue solution was prepared minutes prior to being used, by making 6 vertical cuts on each side of 6, freshly euthanized, donor conspecific fish and then rinsing the fish in 6 ml of seawater. We injected 5 ml of this standard alarm cues solution into the conditioning tanks, giving us a concentration of 2 cuts/L once injected. This concentration has been shown to elicit strong antipredator responses in our test species^25^. The timing of the three injections occurred randomly between 0800 and 1800 h, with a minimum of 1.5 h between consecutive injections.

### Behavioural assays

This methodology followed established bioassays^34^,^35^. Juvenile damselfish were placed individually in 20-L flow-through tanks (32 × 16 × 16 cm) equipped with sand, a small piece of dead coral as a shelter, an airstone, and a 1.5 m long injection tube used to introduce stimuli into the tank. Each tank was covered on three sides with black plastic to avoid visual transfer of information from surrounding tanks. In addition, a black plastic curtain was hung in front of the tanks to minimize disturbance to the fish by the movement of the observer. Fish were left 24 h to acclimate, after which the behavioural assays started. The assay consisted of two 4-min observation periods separated by a 1-min stimulus injection period. Five min prior to the start of each trial, we injected small quantities of food in the tank (2.5 mL of a solution containing ~250 *Artemia* larvae.mL^−1^), to stimulate activity and create a behavioural choice for juveniles to either forage or take refuge within the coral head. This initial feeding event also removed the possibility of a “feeding frenzy” effect at the start of the bioassay. We injected another 2.5 mL of food and started the 4- min pre-stimulus observation period to assess baseline activity level of the fish. We then injected 20 mL of stimulus (seawater, predator odour, herbivore odour, or almond extract) along with 2. mL of food and started the post-stimulus observation period. During each observation period, we measured (1) the total number of feeding strikes displayed by the fish, regardless of whether they were successful at capturing a food item or not and (2) the total number of lines the fish crossed during the observation period, using the 4 × 4 cm grid drawn on the side of the tank. A line was counted as crossed when the entire body of the fish crossed a line. This behaviour represents a measure of the swimming activity of the fish^36^,^37^. The experimenter was blind to the treatment during the observation. To control for day effects, we tested the same number of fish from each of the treatment group each day. The cues tested differed between the first set of trials (young fish, pre-treatment) and all other trials. All 4 cues (water, predator odour, herbivore odour, and almond extract) were used at first, to document and ascertain the response pattern observed was that of a neophobic individual. For subsequent trials, the timeframe necessary to complete all procedures constrained us to limit our test to two cues only (water and novel predator odour).

Predator and herbivore odour were prepared by placing four adult dottyback, *Pseudochromis fuscus*, and four apogonids, *Apogon doederleini*, in a 60-L flow-through tank. The volume of each tank was decreased to 20 L and the flow-through was turned off 2 h before the cues were used. The flow-through was turned on as soon as the cues were collected. We used 1 mL of McCormick (no relation to the co-author of the study) almond extract diluted in 2 L of seawater. We tested 10–12 fish per group.

### Lateralization assays

To assess the behavioural lateralization of the fish, we used a detour test. The apparatus used in this study was based on a design used previously by Bisazza *et al.*^38^ and Dadda *et al.*^39^. Briefly, it consisted of an opaque Perspex tank (60 × 30 × 15.4 cm), with a runway in the middle (25 × 3 × 12 cm) and at both ends of the runway (3 cm ahead of the runway) an opaque barrier (12 cm long × 12 cm height) was positioned perpendicular to the orientation of the runway. Water in the tank was 6 cm deep. At the start of each trial, a single fish was introduced into the middle of the runway and left for 2 min to become accustomed to the environment. During each trial, fish were gently maneuvered to the starting point of the runway. The fish then swam along the runway until it faced the barrier. Fish then had to make a decision to turn left or right around the barrier. To account for any possible asymmetry in the setup, tests were carried out alternately on the two ends of the runway^38^. To avoid fish taking ‘a familiar route’, the fish entered the runway from a different side from which they exited. Turning was scored by direct observation. The criterion used for scoring was the first turning direction taken by the fish when exiting from the runaway. Ten consecutive tests were conducted for each fish. To avoid changes in water temperature and dissolved oxygen levels, both of which have been found to influence neural function^40^, the tank water was changed every ten trials. Water temperature in the experimental tank was maintained at 27–28 °C.

In order to compare the high- and low-risk groups with respect to their left-right preference in the detour test, we calculated an absolute lateralization index (*L*_A_) according to the following formula^38^: absolute value of [(#right turn - #left turn)/(total # of trials, i.e. 10)*100]. The *L*_A_ index ranges from 0 (an individual that turned in equal proportion to the right and to the left – no bias) to 100 (an individual that turned right in all 10 trials, or left in all 10 trials). *L*_A_ allowed us to compare the strength of the lateralization (irrespective of its direction) among groups at the individual level. We tested 30 fish per treatment group.

### Escape performance assays

This methodology followed that of Allan *et al.*^41^. Fish were placed in a clear-bottom circular glass tank (28 cm diameter, 3 L) filled with fresh seawater and left to acclimate for 30 min. Shallow water depth (10 cm) was used in the experimental tank in order to minimize displacement in the vertical dimension. Water temperature in the experimental arena was maintained at 27 °C (mean ± SD). The arena was illuminated with four 150-W spotlights, placed above the water surface, by the side of the tank. Following the acclimation period, a fast-start response was elicited with the release of a weighted test tube into the arena, in the proximity of the fish. The weight was controlled by a piece of fishing line that was long enough such that only the tip of the test tube touched the surface of the water. To avoid a premature escape response associated with visual stimulation occurring, and to allow calculation of the escape latency, the stimulus was released through a white PVC tube (length 30 cm) suspended above the experimental tank, with the bottom edge at a distance of 10 mm above the water level. A mirror angled at 45 ° was placed below the arena to facilitate video-recording without disturbing the fish. Escape responses were recorded at 600 frames per second (fps; Casio Ex-fh20) as a silhouette from below. A 1-cm line was drawn in the centre of the inner arena to enable calibration for video analysis. Response latency was quantified using Image-J software and is defined as the time interval (s) between the stimulus touching the water surface and the first detectable movement of the fish. We tested 9 fish per group.

### Respirometry assays

We used a 4-chamber resting respirometry setup, consisting of darkened 27.5 mL cylindrical chambers. All chambers were fitted with a fiber optic oxygen probe and immersed in a temperature-controlled 20-L aquarium (40 × 25 × 20 cm) filled with filtered, UV-sterilized and fully aerated seawater. The water temperature was maintained at 27.7 ± 0.2 °C (mean ± SD). Dissolved oxygen concentration within the chambers was recorded at 0.5 Hz with a 4-channel FireSting O_2_ Optical Oxygen Meter (Pyroscience, Aachen, 263 Germany), and a closed-loop recirculation peristaltic pump (Cole-Parmer Masterflex multichannel pump), that ensured continuous mixing of the water inside each of the chambers. The setup used was similar to that of Johansen and Jones^42^.

At the beginning of each trial, the respirometry chambers were filled with temperature-controlled, filtered, and fully aerated seawater. Four fish were caught by hand net in the holding tanks and placed in a 100 ml transport container. After 3 min, each fish was placed directly into one chamber and measures of O_2_ concentration started within 10 s. Rates of maximum O_2_ consumption (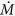
O_2Max_) was calculated from the average reduction in O_2_ concentration during the first 12 min interval. Subsequently, oxygen consumption rates (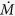
O_2_) were measured continuously following a cycle of 12-min measurement, 9-min flushing and 2-min wait period to replenish the chamber with filtered and oxygenated water. This provided one measure of 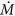
O_2_ every 23 min. An estimation of standard metabolic rate (SMR) was obtained by leaving the fish in the chamber for 8 to 12 hours and averaging the three lowest 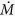
O_2_ values obtained after O_2_ consumption rate stabilized and no longer decreased (equivalent to the lowest 10–15% of measures). The time it took each fish to reach SMR was recorded. After the trial, the fish was removed from its chamber, weighed on a 0.0001 g precision scale and returned to its holding tank. All trials were conducted during daylight hours to avoid any potential differences in diurnal rhythm between individuals. We used a balanced design where two fish from each of the two risk treatments were run simultaneously, with allocation of chambers randomized.

Oxygen consumption rate of individual fish (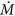
O2 in mg O_2_ kg^−1^ h^−1^) was calculated using LabChart v. 6.1.3 (ADInstruments, Dunedin, New Zealand) as the slope of the linear regression of O_2_ concentration decline over time within each chamber using the equation 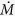
O_2_ = *s*V_resp_αM^−1^ (Bushnell *et al.*, 1994; Schurmann and Steffensen, 1997), where *s* is the slope (mmHg h^−1^), V_resp_ is the volume of the respirometer system including the recirculating loop minus the volume of the fish (L), α is the solubility of O_2_ in water (μgO_2_ L^−1^ mmHg^−1^) adjusted for temperature and barometric pressure and M is the mass of the fish (kg). Background respiration by bacteria in individual chambers was measured before and after each trial, and subtracted from 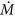
O_2_ values upon calculation. The system was cleaned with 50% ethanol at the end of every trial to ensure that background oxygen consumption rates remained below 30% of the resting metabolic rate of the fish. To ensure accuracy of results, measures of oxygen consumption where discarded when background respiration reached above 30%. Of the 24 fish tested (12 in each treatment), we ended up using 12 individuals from the high-risk and 11 from the low-risk treatments, due to a missing weight measurement of one fish.

### Survival assays

We tested whether high- and low-risk juveniles would differ in survival and whether their survival depended on the type of predator encountered (ambush vs. pursuit), using a well-established protocol^43^. Groups of 4 fish of matching risk treatment (day 1 post-treatment) were placed in outdoor flow-through mesocosm pools (111 cm diameter, 45 cm high, 368 L) containing a 2-cm deep sand substrate, an airstone, and 2 pieces of dead bushy hard coral (*Pocillopora damicornis*) placed beside each other, forming a coral patch of ~90 cm in circumference and ~20 cm in height. The water was pumped directly from the ocean so it followed natural temperature fluctuations. One hour after the introduction of the damselfish, we introduced a single predator (moonwrasse, a pursuit predator, or lizardfish, an ambush predator that typically sits on the bottom covered in sand) in each mesocosm. Both prey and predator were left undisturbed, except for 2 feeding events (1100 and 1700 h), in which we injected 60 mL of a solution of freshly hatched brine shrimp (~250 per mL) in the pool. The next day, all the fish were removed from the pool and we recorded the number of surviving fish. The water was drained, the water flow increased, and the pool reset for the next trial.

One week prior to the start of the experiment, the predators were fed juvenile damselfish, but starved for 24 h prior to being used in a trial. Trials with moonwrasse (11.8 ± 0.2 cm) were paired, in that each predator was used as its own control (n = 16). Moonwrasse were randomly allocated to either the high or low-risk group, in a balanced design. After the end of their first trials, the predators were fed, and then starved for 1 day prior to being used in their second trials, which took place 48 h after their first one. The treatment with which each predator was associated was switched between the first and second trial. For trials with lizardfish (8.7 ± 0.1 cm), some unplanned circumstances prevented us from reusing the lizardfish. We thus carried out 15 trials (n = 7 for low risk, n = 8 for high risk).

### Statistical analysis

#### Question 1: Effect of risk exposure on the expression of a defensive phenotype and survival benefits

A proportion change in behaviour (foraging and activity) from the pre-stimulus baseline was calculated and used a response variable in the analysis. The data for foraging and activity were analyzed together using a MANOVA, testing for the effect of risk (low vs high) and cue (water vs novel predator odour) on the behaviour of the fish (n = 10–12/treatment). For absolute lateralization index, the mean scores between the low- and high-risk groups were compared using an independent t-test (n = 30/treatment). The data for latency to burst were highly heteroscedastic, so the differences between the high and low-risk groups were analyzed non-parametrically with a Mann-Whitney U test (n = 9/treatment). Data on basal metabolic rate and latency to reach rest were analysed using a two-way ANOVA, comparing the values between risk groups (high vs low), and introducing “test day” as a random factor to account for slight variations in temperature among days (n = 11–12/treatment for basal metabolic rate, n = 16/treatment for latency to reach rest). For survival trials, the number of fish surviving in each trial was turned into a proportion of fish surviving (number of fish surviving/total number of fish). A 2-sample paired t-test was used to compare the effect of risk on moonwrasse survival, while an independent-sample t-test was used to compare the survival of the fish to lizardfish. Data met parametric assumptions.

#### Question 2: Ontogenetic effects on the expression of the defensive phenotype

The expression of neophobic tendencies prior to treatment in both settlement-stage (newly collected) and post-settlement fish (10 days post-collection) were assessed by a 2-way MANOVA investigating the effect of ontogeny (settlement vs. post-settlement) and cue (water, predator odour, herbivore odour or almond extract) on the 2 behaviours. To answer the same question using the lateralization endpoint, we carried an independent t-test comparing the degree of lateralization of the settlement-stage vs post-settlement fish. If the timing of risk exposure did not affect the expression of an antipredator phenotype, we would expect a non-significant interaction between age and cue. To investigate any ontogenetic effects on the expression of the defensive phenotypes, we carried a 3-way MANOVA testing the effect of risk (high vs. low), day (1, 8 and/or 15 days post-treatment) and cue (water vs. predator odour) on the behaviour of fish, both settlement-stage and post-settlement (n = 10–12/treatment). We similarly carried a 2-way ANOVA testing the effect of risk (high vs. low), and day (1, 8 and/or 15) on the degree of lateralization expressed by the fish (n = 30/treatment). All parametric assumptions were met. For both age classes, a significant interaction between risk and time would indicate a change in the antipredator response displayed through time. If the defensive phenotype is maintained throughout ontogeny, like is posited for settlement-stage juveniles, we would expect a non-significant interaction between risk and time. However, if the expression of this phenotype wanes through time, a significant interaction should be found.

## Results

### Question 1: Effect of risk exposure on the expression of a defensive phenotype and survival

#### Behaviour

**Figure 2:**
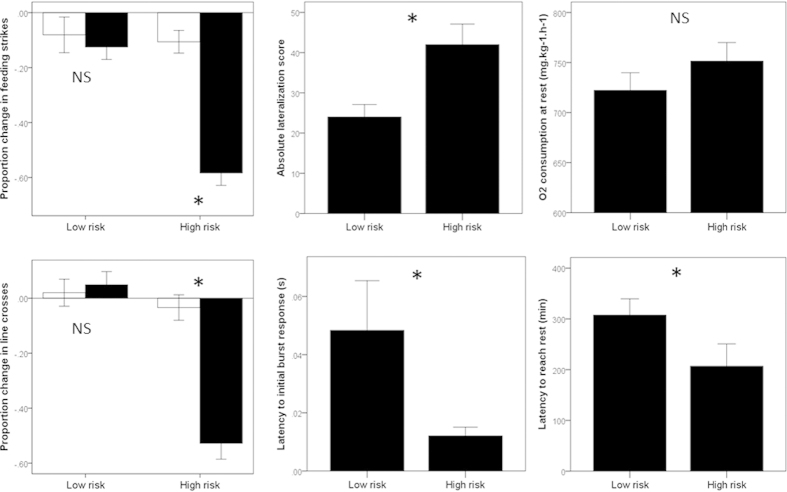
Data summary of all the endpoints used to assess the differences in phenotypes between newly-collected juvenile damselfish (*Pomacentrus chrysurus*) exposed to a low-risk or a high-risk regime for 4 days. Figures represent mean (±SE). For proportion change in feeding strikes and line crosses (left panels), fish were exposed to a seawater control (white bars) or a novel predator odour (black bars). Stars indicate statistical significance at α = 0.05, while “NS” indicates a lack of significant at the same α level.

Groups of fish did not differ in their pre-stimulus behaviour (MANOVA: risk: F_2,38_ = 0.1, P = 0.9; cue: F_2,38_ = 0.3, P = 0.7; interaction: F_2,38_ = 1.6, P = 0.2). We found a significant interaction between risk and cue on the change in behaviour of the fish (MANOVA: F_2,38_ = 13.6, P < 0.001). Fish from the low-risk group did not differ in their response to the two cues (seawater or novel predator odour; P = 0.9), while fish from the high-risk group displayed a significant antipredator response towards the novel predator odour compared to a seawater control (P < 0.001). The response pattern was similar for both response variables (Fig. 2).

#### Lateralization

The t-test revealed that high-risk fish were ~60% more lateralized than low-risk ones (t_58_ = 3.0, P = 0.004, Fig. 2).

#### Latency to burst

The Mann-Whitney U test revealed that high-risk fish responded ~60% faster than low-risk ones (U = 14, P = 0.019, Fig. 2).

#### Physiological data

The 2-way ANOVA failed to find an effect of risk on resting metabolic rate (F_1,16_ = 1.6, P = 0.2), and no effect of day (F_5,16_ = 2.5, P = 0.073). However, risk regime did significantly influence the latency to reach this resting metabolic rate (F_1,23_ = 4.8, P = 0.039, Fig. 2), along with a significant effect of day (F_1,23_ = 2.7, P = 0.036). Fish in the high-risk group reached their resting metabolic rate ~35% faster than those in the low-risk group (Fig. 2).

#### Survival

**Figure 3:**
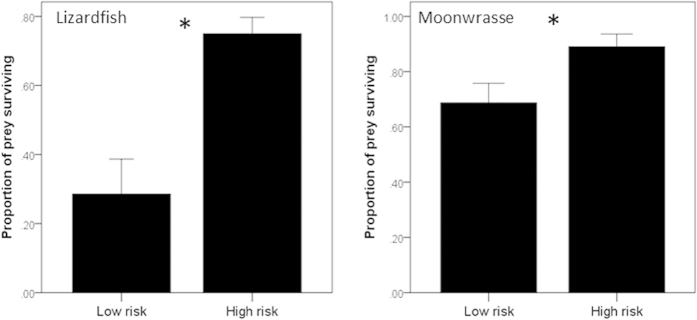
Mean (±SE) proportion of low-risk or high-risk fish surviving during a 22-h predatory stage encounter with either an ambush predator (lizardfish) or a pursuit predator (moonwrasse). Stars indicate statistical significance at α = 0.05.

The t-tests revealed that high-risk fish survived better than low-risk fish, when exposed to lizardfish (independent t-test, t_13_ = −4.3, P = 0.001) and moonwrasse (paired t-test: t_15_ = −2.6, P = 0.018, Fig. 3).

### Question 2: Ontogenetic effects on the expression of the defensive phenotype

#### Comparison prior to treatment

**Figure 4:**
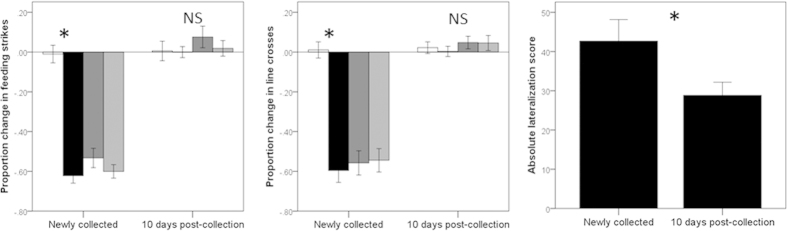
Mean (±SE) proportion change in feeding strikes and line crosses and absolute lateralization index for newly collected fish and fish 10 days after collection. For feeding strikes and line crosses, the fish were exposed to seawater (white bars), a novel predator odour (dottyback, black bars), a novel herbivore odour (apogonid, dark grey bar) or the odour of almond extract (light grey bars). Stars indicate statistical significance at α = 0.05, while “NS” indicates non-significance at the same threshold.

The 2-way MANOVA revealed a significant interaction between age and cue (Pillai’s Trace: F_6,168_ = 11, P < 0.001, Fig. 4) on the behavioural response of the fish. While the response of post-settlement fish did not differ among the different cues (F_6,88_ = 0.4, P = 0.8), we found cue to significantly impact the behaviour of settlement-stage fish (F_6,80_ = 9.6, P < 0.001). Namely, fish responded with a similar intensity to all 3 odours (all P > 0.4), but did not respond to seawater (P < 0.001). These results indicate that settlement-stage fish showed neophobic tendencies, displaying a significant antipredator response to any novel odour, while post-settlement fish maintained in the laboratory for 10 days did not. For the lateralization scores, the settlement-stage fish showed a lateralization score 50% larger than those of older (10-day post-collection) fish (t-test, t_89_ = 2.2, P = 0.026, Fig. 4).

#### Ontogenetic effects

**Figure 5:**
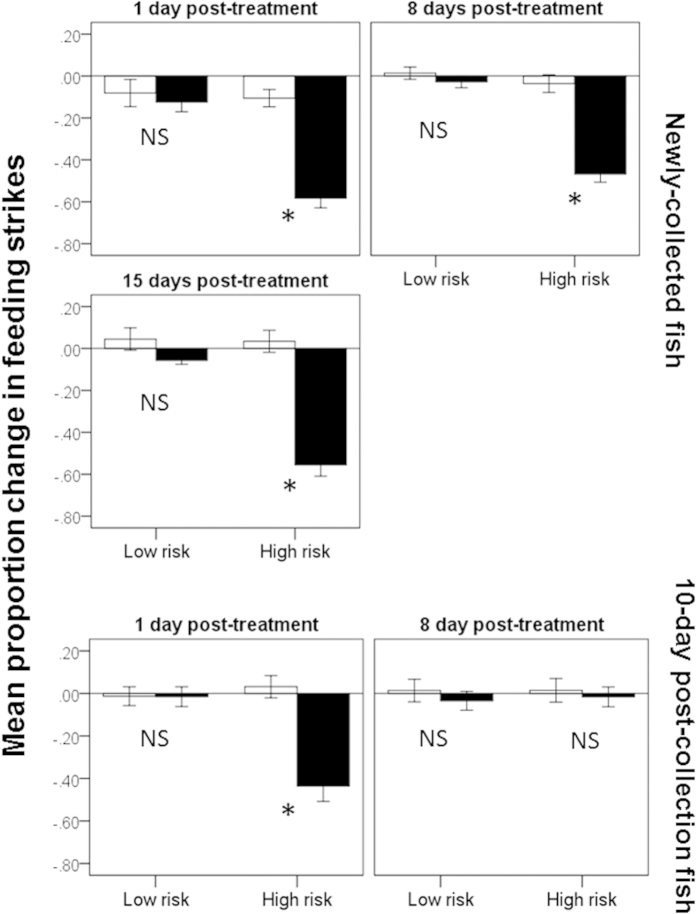
Mean (±SE) proportion change in feeding strikes for newly-collected fish (top panel) or 10-day post-collection fish (bottom panel). The fish were exposed to a low- or high-risk regime for 4 days and tested 1, 8 and/or 15 days post-treatment. Fish were exposed to seawater (white bars) or a novel predator odour (dottyback, black bars). Stars indicate statistical significance at α = 0.05, while “NS” indicates non-significance at the same threshold.

For young fish, the 3-way MANOVA revealed an effect of risk (F_2,123_ = 74.1, P < 0.001), cue (F_2,123_ = 81.7, P < 0.001) and an interaction between the two (F_2,123_ = 50.1, P < 0.001, Fig. 5) on the behaviour of fish. However, we failed to find an interaction between day and risk (F_4,248_ = 0.7, P = 0.6), day and cue (F_4,248_ = 1.8, P = 0.1) and day, risk and cue (F_4,248_ = 1.4, P = 0.2), indicating that the response pattern is similar across days. Namely, fish in the low-risk group did not respond differently to water and predator odour (P = 0.1), while those in the high-risk group displayed an antipredator response to the predator odour (P < 0.001).

For older fish, the 3-way MANOVA revealed a significant interaction among risk, day and cue (F_2,86_ = 10.4, P < 0.001, Fig. 5). When tested one day after treatment, the behaviour of the fish were affected by an interactive effect of risk and cue (F_2,43_ = 12.6, P < 0.001). Fish in the low risk group did not respond differently to water or predator odour (P = 0.6), while those in the high risk group displayed an antipredator response to the predator odour (P < 0.001). On day 8, however, we failed to find an effect of cue (F_2,42_ = 0.5, P = 0.6), risk (F_2,42_ = 0.1, P = 0.9), or any interaction (F_2,42_ = 0.7, P = 0.5) on the behaviour of the fish, indicating that the fish did not respond to the predator odour, regardless of risk level.

**Figure 6:**
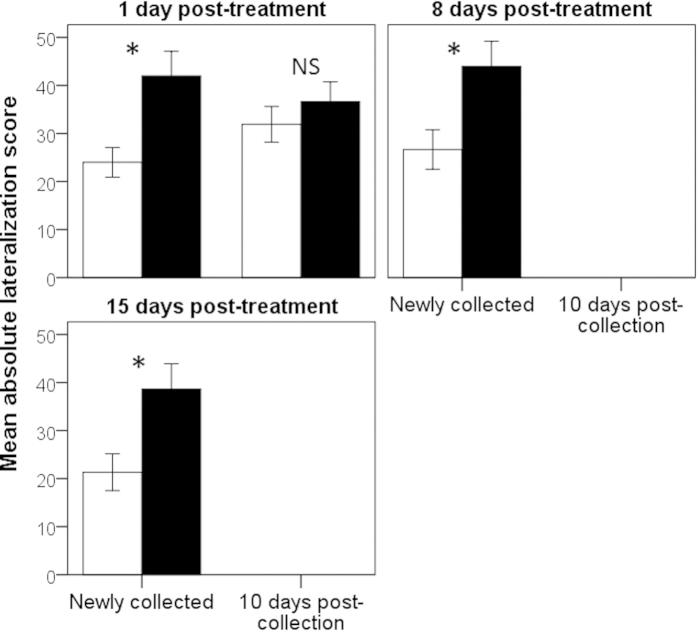
Mean (±SE) absolute lateralization score for fish kept under a low-risk (white bars) or high-risk (black bars) regime for 4 days and tested after 1, 8 or 15 days. Stars indicate statistical significance at α = 0.05, while “NS” indicates non-significance at the same threshold.

For the lateralization data, the 2-way ANOVA performed post-treatment on the newly-collected fish revealed a significant effect of risk (F_1,174_ = 22.8, P < 0.001, Fig. 6), but no effect of day (F_2,174_ = 0.7, P = 0.5) nor any interaction between the two factors (F_1,174_ = 0.1, P > 0.9). This indicates that high-risk fish displayed a greater lateralization score than low-risk fish, and this, for the duration of the 2-week period, without any evidence of waning. For the fish tested 10-day post-collection, however, the results indicated that one day post-treatment, we failed to find a difference in the lateralization score of the 2 groups (F_1,82_ = 0.7, P = 0.4). In fact, the lateralization score of the fish post-treatment did not differ from that of the fish pre-treatment (F_1,142_ = 0.3, P = 0.6).

## Discussion

Our results clearly indicate that exposure to low- vs high-risk environments elicit a pronounced change in the antipredator phenotype of juvenile damselfish. Fish exposed to high background risk conditions exhibit neophobic tendencies in response to novel stimuli and displayed stronger behavioural lateralization scores, when compared to their low-risk counterparts. These results are in line with previously published work^25^,^26^. However, high-risk fish also responded faster to a looming stimulus (i.e., lower latencies), and reached metabolic rest quicker than the low-risk fish, and showed a slight increase in resting metabolic rate, although this increase did not appear significant. Overall, the high-risk group appeared to exhibit more effective escape behaviours compared to fish from low risk. Here, we document that a short exposure to risk can elicit simultaneous changes in a number of physiological and behavioural antipredator-related traits, providing insight into the integration of risk information at the individual level. Our traditional view is that environmental variation needs to be of high intensity or long duration to affect individuals or populations in any significant way. However, recent evidence would suggest that in fact, environmental conditions of weak intensity or short duration could have similar effects if they occur at key stages^44^. This concept of phenotypic resonance^45^ has been documented in lizards, for which the food consumed during their first meal could have measurable consequences months later. Similar to a lizard’s first meal, our data seem to suggest that predation may be a critical determinant of life-history strategies: a few days of high-risk conditions could have a myriad of effects with long-lasting consequences, although we were not able to follow the fish for more than 2 weeks.

How do these traits integrate? These high-risk fish are fearful to anything new at first, as shown by the neophobic tendencies to even biologically-irrelevant stimuli^24^. However, studies looking at the development of neophobia indicate that fish quickly learn to categorize non-risky stimuli as irrelevant^46^. Nevertheless, these fish will likely respond to perceived threats more frequently than their low-risk counterparts. Interestingly, they are able to calm down and reach metabolic rest in half the time of low-risk fish. This likely helps lower the costs associated with frequent responses. When exposed to a looming stimulus, high-risk fish respond much quicker. Fish escape responses have been linked to either Mauthner (or M-)cells responses, which is responsible for a reflex-like escape in the direction opposite to the side that detects the stimulus, or non M-cell responses that typically elicit responses after a longer delay^47^,^48^. The difference in latency to respond that we documented could reflect fish responding with different circuits (M-cell vs. non-M-cell responses) or could simply reflect a lower reaction time of the high-risk fish due to their expectations to be attacked. The consequence of these defensive traits is that high-risk fish are more likely to survive predatory attacks by predators^49^, although the magnitude of the benefit seems higher for lizardfish (ambush-type predator), than moonwrasse (pursuit predator). Whether increased survival results from the expression of a single trait or a combination of all traits is still to be determined. However, we do not know whether those traits can be decoupled from one another.

Few studies have examined how prey can integrate multi-level antipredator defences to decrease their risk of predation^50^,^51^,^52^,^53^,^54^. In one such study, Dewitt *et al.*^51^ considered the integration of behavioural and morphological defences in snails, finding that narrow-aperture snails with heavily-defended shells showed a weaker behavioural responses to predators (trait compensation), while others with narrow aperture would show a positive correlation between morphological and behavioural investment (trait co-specialization). In the current study, the expressions of behavioural and physiological traits were initiated by the exposure to risk, which is similar to Dewitt’s trait co-specialization: all traits are necessary for effective antipredator defences. Neophobic tendency may be important early in the predation sequence, as the prey may try to avoid an encounter with a novel predator^24^. Both behavioural lateralization and latency to respond play a role later in the predation sequence, when the predator has detected and attacked the prey^39^,^55^,^56^. The ability to reach metabolic rest quicker may allow prey to resume foraging and other fitness-related activities quicker, and minimize the costs of responding.

The second part of our study provides evidence supporting the idea that the expression of this defensive phenotype is in some ways linked to the ontogeny of the fish. Similar risk treatments that are temporarily offset clearly lead to different response patterns: settlement-stage fish (1-day post-collection) are highly responsive to the risk-treatment, with a 4-day exposure leading to behavioural effects lasting at least 2 weeks post-treatment, without waning of the response intensity. An identical treatment received by post-settlement fish (10 days post-collection) led to a limited expression of the defensive phenotype (neophobic tendencies were affected by the treatment, but lateralization was not), and this expression, when present, was short-lived. The neophobia effects were not present one week after the end of the treatment. We have to use caution in the interpretation of the results. Because of the temporal randomization of the experiment, the results cannot be explained by a temporal bias. However, the post-settlement fish spent 10 days in the laboratory, in the absence of any predator-related cues. We think it is this safe environment at a key transition that is causing a decrease in the responsiveness of the fish to future risk exposure. We do not think our results reflect a laboratory bias because both groups of fish were exposed to the same housing conditions (temperature, food, light, social environment). In addition, our post-settlement fish were healthy, in good body condition and they displayed neophobic tendencies to a level similar to that of the settlement-stage fish (~50% reduction in activity). The potential existence of a critical period for the development of an integrated antipredator phenotype in prey could have important implications for conservation. Reintroduction programs often rely on training to get captive naïve individuals ready for reintroduction^57^. If such training does not occur at the right time, the animal may display a suboptimal antipredator response. It is unknown if experience can compensate for missed exposure to risk during the critical period.

While our results provide evidence of considerable benefits associated with an integrated anti-predator phenotype, we need to consider that the expression of a defensive phenotype is likely to be costly^58^,^59^,^60^. Neophobic tendencies in a novel, high-risk environment are plastic, indicating that the costs would override the benefits in a low(er)-risk environment. Prey with a higher degree of lateralization are thought to be at an advantage when responding to predator attacks. However, the existence of prey with a low degree of lateralization implies, once again, an inherent cost^61^. The cost of a change in metabolic parameters is not immediately apparent, but plasticity suggests that changes may be costly. Prey with a defensive phenotype should be at a selective advantage if the survival benefits override the costs. How much predation pressure is needed to override these costs is a fascinating topic for future work. Likewise trying to understand whether or not the expression of this defensive phenotype is graded or simply an all-or-nothing phenomenon is unknown. Preliminary evidence suggests that the intensity or duration of neophobia can be modulated by the level of risk of the environment^46^. Whether the other parameters exhibit a similar pattern with ontogeny requires further research.

## Additional Information

**How to cite this article**: Ferrari, M. C.O. *et al.* Living in a risky world: the onset and ontogeny of an integrated antipredator phenotype in a coral reef fish. *Sci. Rep.* **5**, 15537; doi: 10.1038/srep15537 (2015).
